# Marine mimivirus relatives are probably large algal viruses

**DOI:** 10.1186/1743-422X-5-12

**Published:** 2008-01-23

**Authors:** Adam Monier, Jens Borggaard Larsen, Ruth-Anne Sandaa, Gunnar Bratbak, Jean-Michel Claverie, Hiroyuki Ogata

**Affiliations:** 1 Structural and Genomic Information Laboratory, CNRS-UPR 2589, IBSM, Parc Scientifique de Luminy, 163 avenue de Luminy, Case 934, 13288 Marseille Cedex 9, France; 2 Department of Biology, University of Bergen, PO Box 7800, N-5020 Bergen, Norway

## Abstract

**Background:**

*Acanthamoeba polyphaga *mimivirus is the largest known ds-DNA virus and its 1.2 Mb-genome sequence has revealed many unique features. Mimivirus occupies an independent lineage among eukaryotic viruses and its known hosts include only species from the *Acanthamoeba *genus. The existence of mimivirus relatives was first suggested by the analysis of the Sargasso Sea metagenomic data.

**Results:**

We now further demonstrate the presence of numerous "mimivirus-like" sequences using a larger marine metagenomic data set. We also show that the DNA polymerase sequences from three algal viruses (CeV01, PpV01, PoV01) infecting different marine algal species (*Chrysochromulina ericina*, *Phaeocystis pouchetii*, *Pyramimonas orientalis*) are very closely related to their homolog in mimivirus.

**Conclusion:**

Our results suggest that the numerous mimivirus-related sequences identified in marine environments are likely to originate from diverse large DNA viruses infecting phytoplankton. Micro-algae thus constitute a new category of potential hosts in which to look for new species of *Mimiviridae*.

## Background

The discovery of *Acanthamoeba polyphaga *mimivirus was a significant breakthrough in the recent history of virology. Both mimivirus particle size (~750 nm) and its genetic repertoire (1.2 Mb-genome encoding 911 protein coding genes) are comparable to those of many parasitic cellular organisms [1,2]. This giant virus exhibits several genes for translation system components [3], and its particle contains both DNA and RNA molecules [2]. These features both quantitatively and qualitatively challenge the boundary between viruses and cells, and reignited a smoldering debate about the origin of viruses and their role in the emergence of eukaryotes [4-9].

Mimivirus belongs to Nucleocytoplasmic large DNA viruses (NCLDVs) [10]. From its basal position in the phylogenetic trees based on conserved NCLDV core genes [1,2], the new "*Mimiviridae*" family was proposed for mimivirus [11]. NCLDVs now include *Mimiviridae*, *Phycodnaviridae*, *Iridoviridae*, *Asfarviridae *and *Poxviridae*. Mimivirus is the sole member of the *Mimiviridae *family. The lack of known close relatives of mimivirus makes it difficult to build the evolutionary history of its surprising features. Is mimivirus one of many eccentric creatures in nature such as Rafflesia, a parasitic plant in southeastern Asia known for its gigantic flower [12]? Are the mimivirus extraordinary characteristics linked to the origin of eukaryotes [5]? Clearly, appraising the actual biological significance of this exceptional virus requires the isolation and characterization of additional members of the *Mimiviridae *family.

Mimivirus was initially isolated in amoebae sampled from the water of a cooling tower. Following the circumstances of its discovery, mimivirus was suspected to be a causative agent of pneumonia [13]. The presence of antibodies recognizing mimivirus in the sera of patients with community or hospital-acquired pneumonia was reported [14,15]. However, no serological evidence of mimivirus infection was found in hospitalized children in Austria [16] and mimivirus has never been isolated from an infected patient despite numerous attempts. In the laboratory, mimivirus appears to infect only species of *Acanthamoeba *[17]. *Acanthamoeba *are ubiquitous in nature and they have been isolated from diverse environments including freshwater lakes, river waters, salt water lakes, sea waters, soils and the atmosphere [18,19]. Mimivirus relatives might thus exist everywhere.

Ghedin and Claverie identified sequences similar to mimivirus genes in the environmental sequence library from the Sargasso Sea [20]. This strongly suggested the existence of mimivirus relatives in the sea. More recently, we found numerous additional "mimivirus-like" sequences in the much larger metagenomic data set generated by the Global Ocean Sampling Expedition (hereafter referred to as GOS data; [21]) (Monier *et al*., manuscript in preparation). However, the analysis of metagenomic data (i.e. short sequences from unknown and mixed organisms) provides no insights into the hosts susceptible to harbor the putative new species of *Mimiviridae *corresponding to these sequences.

While continually monitoring the new occurrences of mimivirus-like sequences in public databases, we recently noticed that the type B DNA polymerase (hereafter referred to as PolB) sequences of three lytic viruses from Norwegian coastal waters were very similar to the PolB sequence of mimivirus. The three viruses [CeV01 (GenBank accession: ABU23716), PpV01 (ABU23718), PoV01 (ABU23717)] were isolated from diverse marine unicellular algae: *Chrysochromulina ericina*, *Phaeocystis pouchetii *and *Pyramimonas orientalis*, respectively [22,23]. *C. ericina *and *P. pouchetii *are both haptophytes but phylogenetically distant and classified in different orders, i.e. *Prymnesiales *and *Phaeocystales*. *P. pouchetii *forms dense and almost monospecific spring blooms while *C. ericina *thrive in mixed flagellate communities and at cell densities usually not attaining bloom levels [24,25]. *P. orientalis *is a prasinophyte belonging to the green algae. It has a worldwide distribution but the abundance is most often low with no significant contribution to the overall phytoplankton biomass [26,27]. The three algal viruses infecting these phytoplankters have all been classified as phycodnaviruses.

In this report, we first analyzed the distribution of mimivirus-like sequences found in the GOS data and mapped them on the mimivirus genome. We then performed phylogenetic analyses which indicated a very close relationship between the PolB sequences of mimivirus and the three algal viruses (CeV01, PpV01, PoV01), as well as with their homologs from the metagenomic data set.

## Results

**Figure 1:**
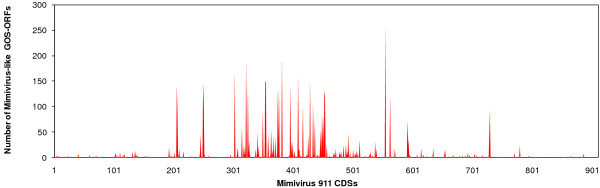
Mimivirus-like sequences in the GOS metagenomic data.

**Figure 2:**
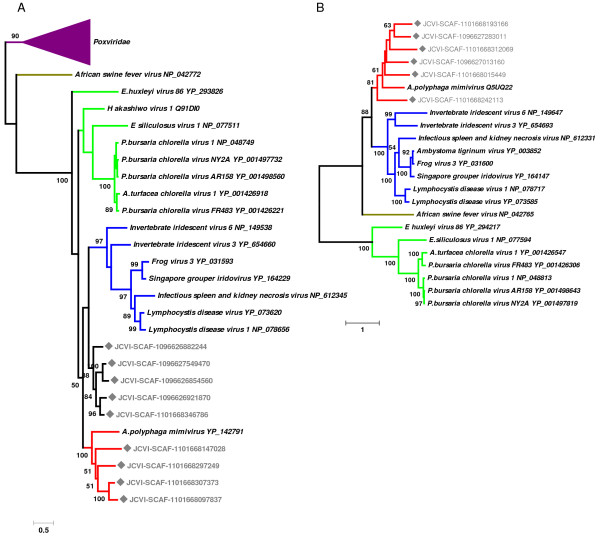
Maximum likelihood trees for two NCLDV class I core genes. (A) Homologs for the mimivirus L437 (VV A32-type virion packaging ATPase). (B) Homologs for the mimivirus L206/L207 (VV D5-type ATPase). Nodes with rectangle marks correspond to the sequences from the GOS data. These trees are unrooted.

We first examined the presence of "mimivirus-like" sequences in the GOS data composed of 7.7 million sequencing reads. Based on a protocol similar to the one used by Ghedin and Claverie [20], we identified 5,293 open reading frames (ORFs; ≥ 60 aa) that are closely related to protein sequences encoded in the mimivirus genome. Of 911 mimivirus protein coding genes, 229 (25%) showed closely related sequences in the GOS data. The distribution of the number of GOS matches for each of the 229 mimivirus genes is highly variable ranging from 1 to 249 (ex. 249 hits for MIMI_R555 DNA repair protein). These 229 mimivirus genes are distributed widely along the chromosome, with an apparently higher concentration in the central part of the genome (Fig. 1). This part of the genome encodes many conserved genes including most of the NCLDV core genes [2]. Mimivirus possesses 26 NCLDV core genes (class I, II and III), of which 17 had close homologs in the GOS data (Table 1 and Additional File 1). Phylogenetic trees for the homologs of two class I core genes (L437, VV A32-type virion packaging ATPase; L206/L207, VV D5-type ATPase) confirmed the separate grouping of the mimivirus sequences with their closest homologs found in the GOS data (Fig. 2) Among the translation related genes of mimivirus, mRNA cap binding protein gene (MIMI_L496) and translation initiation factor eEF-1 gene (MIMI_R624) had close homologs in the GOS data. Remarkably, 55 of the 229 mimivirus genes exhibiting a strong similarity in the GOS data, correspond to ORFans (i.e. ORFs lacking homologs in known species), further suggesting that their GOS homologs belong to viruses closely related to mimivirus.

**Table 1:** A selected list of mimivirus genes with closely related sequences in the GOS data.

**Mimivirus ORF**	**Annotation**	**Number of "mimivirus-like" sequences in the GOS data**
***NCLDV class I core genes***		
MIMI_L206 *	Helicase III/VV D5-type ATPase (C-term)	139
MIMI_L207 *	Helicase III/VV D5-type ATPase (N-term)	90
MIMI_R322	DNA polymerase (B family)	185
MIMI_R350	putative transcription termination factor, VV D6R helicase	90
MIMI_L396	VV A18 helicase	138
MIMI_R400	S/T protein kinase	32
MIMI_L425	Major capsid protein	7
MIMI_L437	VV A32 virion packaging ATPase	71
MIMI_R450	A1L transcription factor	28
MIMI_R596	Thiol oxidoreductase E10R	7
***NCLDV class II core genes***		
MIMI_R339	TFII-like transcription factor	3
MIMI_R493	Proliferating Cell Nuclear Antigen	45
***NCLDV class III core genes***		
MIMI_L244	Rpb2	1
MIMI_L364	SW1/SNF2 helicase (MSV224)	54
MIMI_R382	mRNA Capping Enzyme	189
MIMI_R429	PBCV1-A494R-like, 9 paralogs	145
MIMI_R480	Topoisomerase II	1
MIMI_R501	Rpb1	14
***Translation***		
MIMI_L496	Translation initiation factor 4E, (mRNA cap binding)	11
MIMI_R624	GTP binding elongation factor eF-Tu	3
***DNA repair***		
MIMI_L315	Hydrolysis of DNA containing ring-opened N7 methylguanine	58
MIMI_L359	DNA mismatch repair ATPase MutS	44
MIMI_R406	Alkylated DNA repair	3
MIMI_L687	Endonuclease for the repair of UV-irradiated DNA	2
MIMI_R693	Methylated-DNA-protein-cysteine methyltransferase	9
**Other genes with more than 100 matches**		
MIMI_L250	putative transcription initiation factor IIB	143
MIMI_L251	Lon domain protease	110
MIMI_R303	NAD-dependent DNA ligase	163
MIMI_R325	Metal-dependent hydrolase (Chilo iridescent virus 136R)	136
MIMI_R354	Lambda-type exonuclease	147
MIMI_R355	Unknown	150
MIMI_L375	Unknown	130
MIMI_L377	putative NTPase I	133
MIMI_R409	Unknown	155
MIMI_L434	Unknown	103
MIMI_R453	TATA-box binding protein (TBP)	131
MIMI_L454	Unknown	119
MIMI_R555	putative DNA repair protein	249
MIMI_R563	Contains helicase conserved C-terminal domain (PFAM)	118

* Two ORFs (L206, L207) have been recently merged into a single ORF after the re-sequencing of the genomic region (SWISS-PROT: Q5UQ22, Stéphane Audic, personal communication).

**Figure 3:**
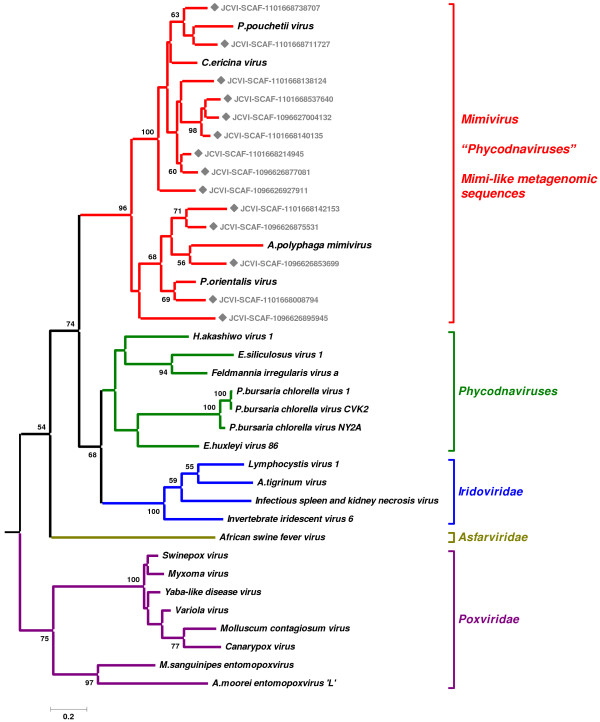
Maximum likelihood tree of the PolB sequences from NCLDV and the GOS data. Nodes with rectangle marks correspond to the sequences from the GOS data. This tree is rooted by phage sequences.

We next selected fourteen mimivirus PolB-like GOS-ORF sequences that are long enough to be fully aligned with homologs from different viruses including three algal viruses, CeV01, PpV01 and PoV01. PolB sequences from CeV01 (GenBank: ABU23716), mimivirus [28] and *Heterosigma akashiwo *virus [29] contain an intein element at the same location. These intein sequences were removed to obtain a canonical multiple alignment of the PolB sequences. This alignment confirmed the conservation of all the known catalytic residues [28] of the polymerase domain. A maximum likelihood tree obtained from the alignment strongly supported the grouping of the mimivirus PolB sequence, its homologs from the metagenomic data and the PolB sequences from CeV01, PpV01 and PoV01 (bootstrap value = 98%; Fig. 3). Similar levels of bootstrap support were obtained by neighbor joining and maximum parsimony approaches (99% and 80%, respectively). Certain of the GOS-ORFs (nine GOS-ORFs) are more closely related to PolB's from CeV01 and/or PpV01 (bootstrap value = 100%), while others appear to be more closely related to PolB's from PoV01 and/or mimivirus. The percentage of identical amino acid residues between mimivirus PolB sequence and its GOS homologs in Figure 3 varies from 37% to 48%, suggesting a substantial level of genetic diversity of the mimivirus relatives in the sea. Mimivirus PolB sequence exhibits 41%, 31%, 45% identity with the PolB sequence of the three algal viruses CeV01, PpV01, and PoV01, respectively. The phylogenetic tree presented in Figure 3 supports the monophyletic grouping for iridoviruses (100%) as well as for poxviruses (75%). In contrast, the inclusion of the new mimivirus-like PolB sequences in the phylogenetic analysis apparently breaks the monophyletic grouping of viruses previously classified as member of the phycodnavirus family, robustly clustering the CeV01, PpV01, and PoV01 viruses with mimivirus.

## Discussion

CeV01, PpV01 and PoV01 were initially isolated from Norwegian coastal waters. An electron cryomicroscopic analysis revealed the icosahedral capsid of PpV01 particles with a maximum diameter of 220 nm [23]. Icosahedral morphology was also suggested for CeV01 (160 nm) and PoV01 (220 × 180 nm) from the observations by transmission electron microscopy [22]. The genomes of these viruses are composed of double-stranded DNA, with estimated sizes being 510-kb for CeV01, 485-kb for PpV01 and 560-kb for PoV01 [22,30]. The genome sizes are substantially larger than the currently sequenced largest phycodnavirus genome (i.e. 407-kb for EhV-86, [31]. Electron microscopy observations of infected cells indicate that viral assembly takes place in the cytoplasm of all three host cells [22,32]. Given these features, these three lytic algal viruses are tentatively classified as phycodnaviruses.

Previous studies have indicated a relatively close phylogenetic relationship [2] and a similarity in gene composition [10] between phycodnaviruses and mimivirus. Several phycodnaviruses exhibit the largest genome sizes (>300-kb) after mimivirus [4]. Claverie *et al*. have hypothesized that *Phycodnaviridae *is a promising source of giant viruses [4]. In this study, we present phylogenetic evidence for a close relationship between the PolB sequences of three algal viruses (CeV01, PpV01, PoV01) and mimivirus, and for the segregation of these from homologs of other known viruses. PolB is one of the NCLDV core genes, and serves as a phylogenetic marker for the classification of large DNA viruses [33,34]. There now seems to be a continuum between the giant mimivirus and some algal viruses at least with respect to the sequence of this essential viral enzyme. The large genome sizes of CeV01, PpV01, and PoV01 might be another indication of their close evolutionary relationship with mimivirus. Phylogenetic classification of phycodnaviruses and mimiviruses (including the split of *Phycodnaviridae *or merging of *Mimiviridae *and *Phycodnaviridae*) may have to be revisited based on sequence information from other genetic markers such as major capsid proteins (Larsen *et al*. manuscript in preparation) and other NCLDV core genes.

Our discovery of the close relationships among PolB sequences of mimivirus and the three algal viruses as well as their homologs from metagenomic data now sheds new light on the nature of the mimivirus relatives in the sea. The mimivirus-like sequences in the metagenomic data are likely to originate from large DNA viruses closely related to mimivirus, CeV01, PpV01 and PoV01. Probably, there is a substantial genetic variation among these putative viruses. The fact that the host algae of CeV01, PpV01 and PoV01 have worldwide distributions, suggests that these putative viruses might not be necessarily associated with marine amoebae, but rather to algal species closely related to *C. ericina*, *P. pouchetii *or *P. orientalis*.

Mimivirus was proposed to be a human pathogen causing pneumonia. However, the close relationship of mimivirus with viruses infecting phytoplankton does not favor this hypothesis, as eukaryotic large DNA virus groups (e.g. at the level of genus) usually correspond to a relatively narrow hosts range. Given the strong cytopathic effect of mimivirus on its amoebal host and its phylogenetic affinity with certain algal viruses, we now begin to suspect that the natural reservoir of mimivirus might be some algae. Indeed, algae are frequently found together with acanthamoeba, in anthropogenic ecosystems such as air-conditioning units.

If horizontal transfer of viral PolB genes does occur, it would become difficult to interpret the PolB phylogeny as representing the true relationships between viruses. However, to the best of our knowledge, no instance of lateral transfer of PolB genes between distantly related eukaryotic large DNA viruses has been documented. The determination of the whole genome sequences of CeV01, PpV01 and PoV01 would definitely help clarifying their evolutionary relationship with mimivirus.

## Conclusion

Three algal viruses (CeV01, PpV01 and PoV01) possess DNA polymerase genes that are closely related to the DNA polymerase from the giant mimivirus. This suggests that the numerous "mimivirus-like" sequences detected in marine metagenomic data might originate from viruses infecting phytoplankton species related to *C. ericina*, *P. pouchetii *or *P. orientalis*, rather than marine amoebae. These results imply new approaches in attempting the isolation of additional, and eventually closer, relatives of mimivirus.

## Methods

The scaffold sequences for the combined assembly of the GOS metagenomic data were downloaded from the CAMERA web site [35]. We extracted 21,406,171 ORFs (≥ aa) from the scaffolds using the EMBOSS/getorf program [36].

We defined "mimivirus-like ORFs" based on the following two-way BLASTP searches [37]. First, the amino acid sequences of the ORFs were searched against the UniProt sequence database release 11.3 (as of July 2007, [38]) using BLASTP (E-value < 0.001). This search resulted in 6,212 ORFs with its best hit to a mimivirus protein in the database. For each of the 6,212 ORFs, we extracted a segment of the mimivirus sequence that was aligned with the ORF by BLASTP. Next, this partial mimivirus sequence was searched against the UniProt database (excluding mimivirus entries in the database). If the best score obtained by this second BLASTP search is lower than the BLASTP score obtained by the first BLASTP search, we kept the ORF as "mimivirus-like". Accordingly, we obtained 5,293 mimivirus-like ORFs. The UniProt database does not contain the three entries used for the phylogenetic study (i.e. ABU23716, ABU23717, ABU23718).

Mimivirus ORFans were defined by the lack of detectable homologs in the UniProt database using BLASTP with an E-value threshold of 0.001.

Multiple sequence alignment was constructed using MUSCLE [39]. All the gap-containing sites in the alignment were excluded in the phylogenetic analysis. We used only the polymerase domain sequences, and removed exonuclease domain sequences. The delineation of the polymerase domains were performed using the Pfam entry PF00136 [40]. Intein sequences were also removed from Mimivirus, HaV, CeV01 PolB sequences. Maximum likelihood phylogenetic analysis was performed using PhyML [41] with JTT substitution model and 100 bootstrap replicates. Neighbor joining analysis was performed using BIONJ [42]. The above methods are available from the Phylogeny.fr server [43]. Maximum parsimony analysis was performed using PHYLIP/PROTPARS [44].

## List of abbreviations used

CeV: *Chrysochromulina ericina *virus; PpV: *Phaeocystis pouchetii *virus; PoV: *Pyramimonas orientalis *virus; NCLDV: Nucleocytoplasmic large DNA virus; GOS: Global Ocean Sampling Expedition; PolB: type B DNA polymerase; ORF: open reading frame.

## Competing interests

The author(s) declare that they have no competing interests.

## Authors' contributions

AM performed the phylogenetic analyses. JBL and RAS contributed new sequence data. HO performed the analyses of the metagenomic data set. GB, JMC and HO contributed to the writing of the manuscript. All authors have read and approved the final document.

## Supplementary Material

Additional file 1 Number of Mimivirus-like sequences in the GOS metagenomic data set. The file shows the number of "mimivirus-like" ORFs that we found in the GOS metagenomic data set for each mimivirus ORF. Click here for file
